# Incidence Trends of Vulvar Cancer in the United States: A 20‐Year Population‐Based Study

**DOI:** 10.1002/cnr2.2120

**Published:** 2024-06-21

**Authors:** Seyed Ehsan Mousavi, Hoomaan Ghasemi, Morvarid Najafi, Zahra Yekta, Seyed Aria Nejadghaderi

**Affiliations:** ^1^ Neurosciences Research Center, Aging Research Institute Tabriz University of Medical Sciences Tabriz Iran; ^2^ Department of Community Medicine, Faculty of Medicine, Social Determinants of Health Research Center Tabriz University of Medical Sciences Tabriz Iran; ^3^ Center for Orthopedic Trans‐Disciplinary Applied Research Tehran University of Medical Sciences Tehran Iran; ^4^ Calaveras County Department of Health Calaveras County California USA; ^5^ HIV/STI Surveillance Research Center, and WHO Collaborating Center for HIV Surveillance, Institute for Futures Studies in Health Kerman University of Medical Sciences Kerman Iran; ^6^ Systematic Review and Meta‐analysis Expert Group (SRMEG) Universal Scientific Education and Research Network (USERN) Tehran Iran

**Keywords:** cancer, epidemiology, incidence, SEER, Surveillance, Epidemiology, and End Results, United States, vulvar neoplasms

## Abstract

**Background:**

Despite being uncommon, vulvar cancer is a serious health concern with a 5‐year relative survival rate of 71%.

**Aims:**

The objective was to investigate the incidence rates of this disease across different racial, ethnic, and histological subgroups in the United States, as well as the effects of the COVID‐19 pandemic on this cancer.

**Methods:**

We used the Surveillance, Epidemiology, and End Results (SEER) 22 database. The International Classification of Diseases for Oncology Version 3 topologic code C51 was assigned for vulvar cancer. Average annual percent change (AAPC) and the pairwise comparison with the parallelism and coincidence were reported. Counts and age‐adjusted incidence rates per 100 000 individuals with their corresponding 95% confidence intervals (CIs) were reported.

**Results:**

The age‐adjusted incidence rate of vulvar cancer was 2.40 (95% CI, 2.38–2.43) over the period 2000–2019, with an AAPC of 0.80 (95% CI, 0.63–0.99). By race/ethnicity, only non‐Hispanic Whites had an increasing trend over 2000–2019 (AAPC: 1.30; 95% CI, 1.07–1.54). The highest age‐adjusted incidence rate of vulvar cancer in the United States was for squamous cell carcinoma (SCC). There was a significant decrease in the age‐standardized incidence rate of vulvar cancer in all races/ethnicities in all age groups (AAPC: −10.15; 95% CI, −15.35 to −4.94) over 2019–2020. Also, the incidence rates and incident numbers of vulvar cancer increased with aging and peaked at the 85+ age group.

**Conclusion:**

There was an increase in the incidence rate of vulvar cancer in all races, with a significantly increased trend in non‐Hispanic White women from 2000 to 2019. SCC displayed the highest incidence rate among vulvar cancer histological types. It is recommended to conduct further research to identify the relevant risk factors of vulvar cancer in the United States.

## 1 Introduction

Vulvar cancer is a relatively uncommon gynecological neoplasm, representing approximately 5%–8% of all gynecological cancers in the United States [1]. It accounts for 0.6% of women's cancers in the United States and primarily affects older women [2]. Every year in the United States, there are around 6470 new cases of vulvar cancer and approximately 1670 attributable deaths, with a 5‐year relative survival rate of 71%, which makes it a significant health concern [3, 4]. Almost 75% of vulvar cancer cases comprise squamous cell carcinoma (SCC), the most prevalent histologic type [5]. A study of the Dutch Cancer Registry conducted between 1989 and 2010 showed that the typical distribution of histologies was the highest for SCC (81%) and basal cell carcinoma (BCC) (8%) [5]. However, other studies have found other histologies more prevalent than BCC such as malignant melanoma or invasive Paget's disease [6].

Over the last two decades, the incidence of vulvar cancer has risen in the United States and other high‐income regions. One study in the United States reported a consistent 1.2% annual rise in human papillomavirus (HPV)‐associated vulvar cancer cases [2]. Another study spanning from 2001 to 2018 noted an increase in overall vulvar cancer rates from 1.83 to 1.96 per 100 000 individuals [7].

Extensive epidemiological data indicate that race or ethnicity correlates with variations in cancer occurrence and fatality rates [8]. The latest research indicates that the highest overall cancer incidence is observed in White individuals, closely followed by American Indian/Alaska Native and Black individuals. A study of 75 767 participants in the United States showed that Whites exhibited the highest average annual percentage increase, followed by Blacks, Asian/Pacific Islanders, and Hispanics for vulvar cancer [9]. This study also evaluated vulvar cancer incidence based on its common histology types, reporting a rate of 77.7%, 6.94%, 5.31%, 1.15%, and 8.87% regarding SCC, adenocarcinoma, melanoma, sarcoma, and other histologies, respectively [9]. Recognizing cancer incidence trends is instrumental in informing public health policies, clinical practices, and research initiative. Given its low incidence and various histological types, the data on vulvar cancer are scarce. With the constantly changing incidence rates of vulvar and other types of cancers, there is a need for extensive and more recent knowledge of this cancer's epidemiological trends. These data will be particularly useful in designing and implementing targeted prevention and intervention programs for susceptible populations. Previously, the vulvar cancer incidence was reported using the preceding iterations of the Surveillance, Epidemiology, and End Results (SEER) database in different time periods, including 1998–2003 [10], 1973–2010 [11], 2001–2017 [9], and 2001–2018 [7]. Furthermore, prior research has not placed primary emphasis on vulvar cancer as their central subject [12, 13], and those studies that have done so, have generally focused on previous version of the SEER database, did not report methodological elements such as the parallelism and coincident tests, and did not evaluate the incidence rates after the COVID‐19 pandemic [7, 9, 14]. Therefore, this paper aimed to identify patterns and trends in the incidence rates of vulvar cancer across demographically and morphologically variant subgroups in the United States in a 20‐year time period by using the SEER database. Moreover, the effects of the COVID‐19 pandemic on the incidence trends of vulvar cancer were investigated.

## 2 Methods

### 2.1 Study Design and Data Source

It is a retrospective, population‐based observational study. The SEER Program of the National Cancer Institute (NCI) is the only comprehensive population‐based information source in the United States that provides information on patient survival rates as well as cancer stage at the time of diagnosis. The SEER 22 covers almost 48% of the US population [15]. The SEER program routinely gathers information on the patient's demographics, primary tumor site, tumor morphology, stage at diagnosis, initial course of therapy, and vital status follow‐up [15]. The current study, used the SEER 22 database to estimate the incidence rates and annual percent changes (APCs) of microscopically diagnosed vulvar cancer from 2000 to 2020 [16]. The SEER 22 database was accessed under the SEER Research Data Agreement for the 1975–2020 Data (November 2022 Submission) [17].

### 2.2 Definitions

Cancer cases are reported as frequencies and percentages, and the incidence rate is expressed as cases per 100 000 population. The APCs of vulvar cancer for a dedicated period show the variation of rates at a constant proportion of the preceding year's rate. The average annual percent changes (AAPCs) describe the average of multiple APCs over the period. The race and ethnicity were classified as non‐Hispanic White, non‐Hispanic Black, and Hispanic, which includes White Hispanic and Black Hispanic patients. Because of the low sample size, the race and ethnicity groups of American Indian/Alaskan Native, Native Hawaiian, and Asian/Pacific Islander cases were only used to calculate the parameters of all races and ethnicities. Patients with vulvar cancer were identified based on the International Classification of Diseases for Oncology Version 3 code C51. The morphologies of vulvar cancer were classified as SCC (codes 8050–8089), BCC (codes 8090–8110), adenocarcinoma (codes 8140–8389), and other morphologies (codes other than 8140–8389, 8050–8089, and 8090–8110).

### 2.3 Statistical Analysis

The SEER 22 database for 2000–2020 [2] was obtained from SEER*Stat, Version 8.4.1.2 [18]. The case selection was set to include only females with microscopically diagnosed vulvar cancer and known age; then, the included cases were stratified based on race, ethnicity, and morphology. The SEER*Stat Version 8.4.1.2 [18] was used to estimate the age‐standardized incidence rates based on the 2000 United States standard population and the associated 95% confidence intervals (CIs) with the Tiwari method [19]. The Joinpoint Regression Program, Version 5.0.2 [20], was used to estimate the APCs, AAPCs, Joinpoint Regression modeling, parallelism test, and coincident test for age‐standardized incidence rates [21]. The year 2020 was the first year of the COVID‐19 pandemic, leading to decreases in cancer screening and diagnosis, resulting in a fall in the majority of cancer sites' 2020 incidence rates [22]. As a result, the 2020 incidence data may introduce bias into cancer incidence estimates; hence, it was omitted from Joinpoint trends and only displayed in illustrations. The APCs of vulvar cancer age‐standardized incidence rates were estimated by generating the best fit of least‐squares regression lines on the natural logarithm of the age‐standardized incidence rate, setting the year of diagnosis as a regressor variable. The minimal number of observations between two Joinpoints and from the Joinpoint to either end of the data was set to two. For model selection, the permutation test with a total significance level of 0.05 and 4 499 permutations was used [21]. The 95% CIs of APCs and AAPCs were calculated using a parametric method to determine whether the APCs and AAPCs were statistically different from zero. The pairwise comparison with the parallelism test was performed to examine whether the trends of the two groups over time were similar [23]. Also, the pairwise comparison with the coincidence test was conducted to investigate whether the rates of the two groups over time were identical.

## 3 Results

### 3.1 Overall Incidence Trends

**FIGURE 1:**
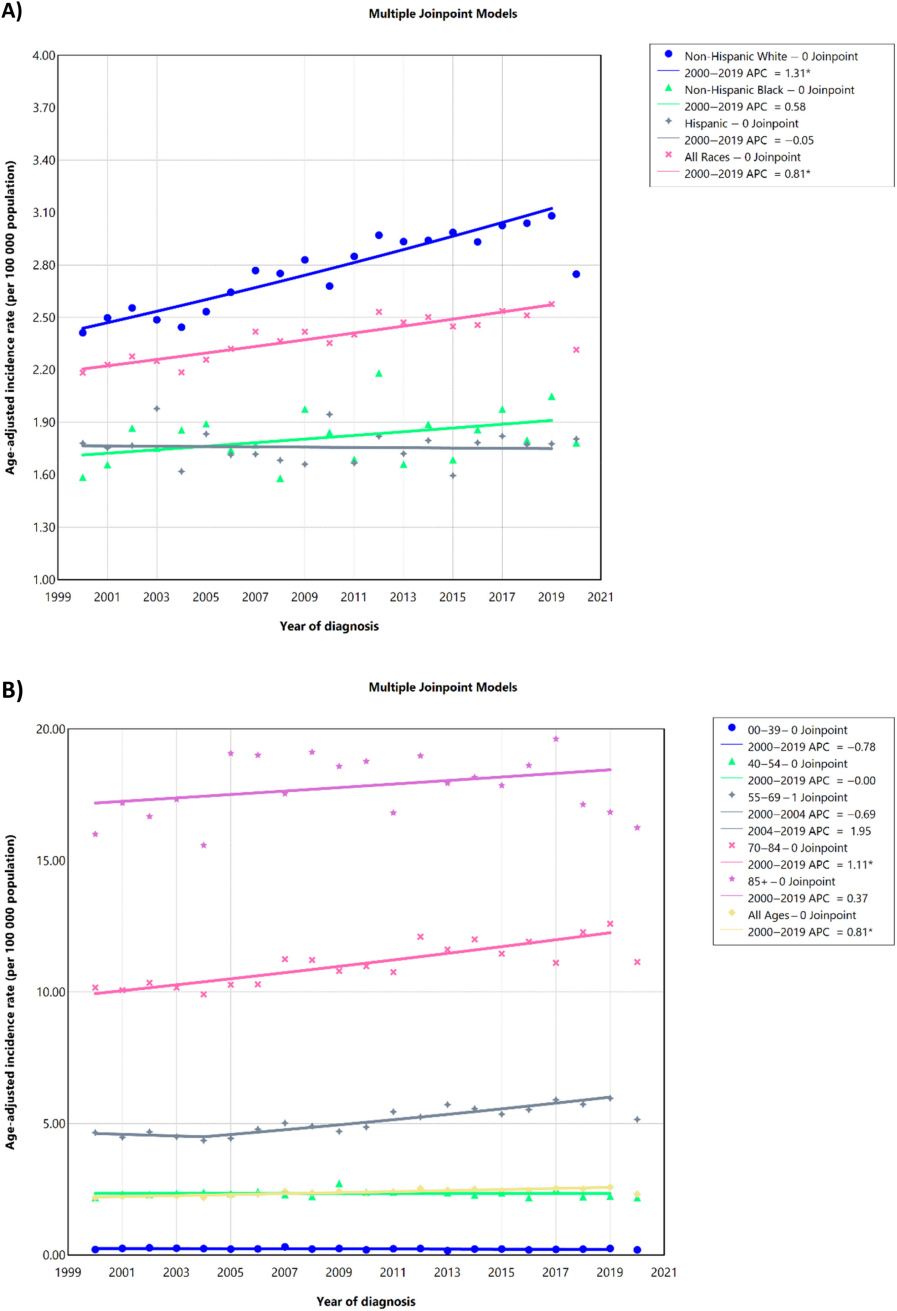
Age‐adjusted incidence rate of vulvar cancer over 2000–2019 and in 2020 in the United States, by race/ethnicity (A) and age (B). *p < 0.05. APC, annual percent change.

A total of 42 352 cases of vulvar cancer were reported in the United States between 2000 and 2020. The majority were non‐Hispanic Whites (*n* = 33 499; 82.12%), aged 70–84 years (*n* = 14 029; 33.12%), living in urban areas (*n* = 36 322; 85.76%), and with median income ≥$65 000 per year (*n* = 25 298; 59.73%). Moreover, the reporting source was mostly hospital inpatient or outpatient records (*n* = 36 642; 86.52%) (Table 1). In 2019, the overall age‐adjusted incidence rate was 2.40 (95% CI, 2.38–2.43) over 2000–2019, with an AAPC of 0.80 (95% CI, 0.63–0.99) (Table 2). Over 2000–2019, there was an increasing trend of the age‐adjusted incidence rate of vulvar cancer in the United States by race/ethnicity (Figure 1A) and by age (Figure 1B). Data S1 represents the overall parallel and identical trends of vulvar cancer.

**TABLE 1:** Demographic characteristics of vulvar cancer cases by the year of diagnosis.

Characteristics	2000–2004, *N* (%)	2005–2009, *N* (%)	2010–2014, *N* (%)	2015–2019, *N* (%)	2000–2019, *N* (%)	2000–2020, *N* (%)
Age (years)						
00–39	482 (5.90)	474 (5.07)	413 (3.88)	446 (3.74)	1815 (4.53)	1894 (4.47)
40–54	1747 (21.40)	1939 (20.75)	1958 (18.40)	1821 (15.29)	7465 (18.63)	7796 (18.41)
55–69	1948 (23.86)	2460 (26.33)	3322 (31.22)	3985 (33.45)	11 715 (29.24)	12 462 (29.42)
70–84	2846 (34.86)	3036 (32.49)	3362 (31.60)	3962 (33.26)	13 206 (32.97)	14 029 (33.12)
85+	1141 (13.98)	1435 (15.36)	1585 (14.90)	1698 (14.25)	5859 (14.63)	6171 (14.57)
Median income per year^a^						
<$35 000	51 (0.62)	57 (0.61)	102 (0.96)	96 (0.81)	306 (0.76)	311 (0.73)
$35 000–$49 999	536 (6.57)	829 (8.87)	1271 (11.95)	1069 (8.97)	3705 (9.25)	3880 (9.16)
$50 000–$64 999	2063 (25.27)	2811 (30.08)	4120 (38.72)	3313 (27.81)	12 307 (30.72)	12 858 (30.36)
≥$65 000	5510 (67.49)	5646 (60.42)	5147 (48.37)	7434 (62.41)	23 737 (59.25)	25 298 (59.73)
Other^b^	4 (0.05)	1 (0.01)	0 (0.00)	0 (0.00)	5 (0.01)	5 (0.01)
Area of residence						
Urban	7035 (86.17)	7970 (85.30)	9137 (85.87)	10 199 (85.62)	34 341 (85.72)	36 322 (85.76)
Rural	1123 (13.76)	1367 (14.63)	1493 (14.03)	1709 (14.35)	5692 (14.21)	6000 (14.17)
Other^c^	6 (0.07)	7 (0.07)	10 (0.09)	4 (0.03)	27 (0.07)	30 (0.07)
Race/ethnicity						
Hispanic	636 (8.02)	784 (8.66)	1024 (9.98)	1230 (10.80)	3674 (9.51)	3959 (9.70)
NHB	639 (8.06)	728 (8.05)	845 (8.24)	937 (8.22)	3149 (8.15)	3336 (8.18)
NHW	6653 (83.92)	7536 (83.29)	8388 (81.78)	9226 (80.98)	31 803 (82.34)	33 499 (82.12)
Reporting source						
Autopsy only	1 (0.01)	0 (0.00)	2 (0.02)	0 (0.00)	3 (0.01)	3 (0.01)
Death certificate only	43 (0.53)	44 (0.47)	67 (0.63)	82 (0.69)	236 (0.59)	248 (0.59)
Hospital inpatient/outpatient or clinic	7376 (90.35)	8224 (88.01)	9119 (85.70)	10 052 (86.80)	34 771 (86.80)	36 642 (86.52)
Laboratory only (hospital or private)	179 (2.19)	268 (2.87)	358 (3.36)	407 (3.42)	1212 (3.03)	1328 (3.14)
Nursing/convalescent home/hospice	10 (0.12)	10 (0.11)	15 (0.14)	12 (0.10)	47 (0.12)	47 (0.11)
Other hospital outpatient unit or surgery center	327 (4.01)	454 (4.86)	705 (6.63)	884 (7.42)	2370 (5.92)	2570 (6.07)
Physician's office/private medical practitioner	207 (2.54)	199 (2.13)	200 (1.88)	207 (1.74)	813 (2.03)	860 (2.03)
Radiation treatment or medical oncology center	21 (0.26)	145 (1.55)	174 (1.64)	268 (2.25)	608 (1.52)	654 (1.54)

Abbreviations: NHB, non‐Hispanic Black; NHW, non‐Hispanic White.

^a^ Median household income adjusted to 2021 inflation.

^b^ Unknown, missing, and no match.

c Unknown, missing, no match, and Alaska or Hawaii.

**TABLE 2:** Rate of vulvar cancer in 2019 and from 2000 to 2019 in the United States and the average annual percent change in rates per 100 000, by race/ethnicity and cancer histology.

		Overall		Non‐Hispanic White		Non‐Hispanic Black		Hispanic	
		Rate over 2000–2019 (95% CI)	AAPC in rate 2000–2019 (95% CI)	Rate over 2000–2019 (95% CI)	AAPC in rate 2000–2019 (95% CI)	Rate over 2000–2019 (95% CI)	AAPC in rate 2000–2019 (95% CI)	Rate over 2000–2019 (95% CI)	AAPC in rate 2000–2019 (95% CI)
	Incidence‐overall	2.4 (2.38 to 2.43)	0.8 (0.63 to 0.99)	2.79 (2.76 to 2.82)	1.3 (1.07 to 1.54)	1.82 (1.76 to 1.89)	0.58 (−0.27 to 1.53)	1.76 (1.7 to 1.82)	−0.02 (−0.46 to 0.51)
Histologic type	Adenocarcinoma	0.04 (0.04 to 0.04)	−0.55 (−2.14 to 1.12)	0.04 (0.04 to 0.04)	−1.17 (−2.96 to 0.63)	0.05 (0.04 to 0.06)	1.15 (−3.23 to 7.03)	0.04 (0.03 to 0.04)	0.23 (−3.81 to 5.38)
	SCC	1.82 (1.8 to 1.84)	1.08 (0.86 to 1.33)	2.13 (2.11 to 2.16)	1.66 (1.28 to 2.12)	1.49 (1.43 to 1.55)	0.75 (−0.08 to 1.66)	1.31 (1.26 to 1.37)	−0.11 (−0.53 to 0.39)
	BCC	0.15 (0.15 to 0.16)	−0.46 (−1.37 to 0.49)	0.17 (0.16 to 0.18)	−0.54 (−1.53 to 0.46)	0.06 (0.05 to 0.07)	0.56 (−4.4 to 6.74)	0.12 (0.1 to 0.13)	0.45 (−1.87 to 3.36)
	Other	0.39 (0.38 to 0.4)	0.12 (−0.39 to 0.66)	0.44 (0.43 to 0.46)	−0.12 (−0.66 to 0.74)	0.22 (0.2 to 0.25)	−1.2 (−3.67 to 1.48)	0.29 (0.27 to 0.31)	−0.16 (−1.99 to 2.12)

Abbreviations: AAPC, average annual percent change; BCC, basal cell carcinoma; CI, confidence interval; SCC, squamous cell carcinoma.

### 3.2 Incidence Trends by Race and Ethnicity

Non‐Hispanic Whites had the highest overall incidence rate of 2.79 (95% CI, 2.76–2.82) over the 2000–2019 period. This upward trend was further supported by an AAPC of 1.30 (95% CI, 1.07–1.54), indicating a significant rise in incidence rates during these years. Non‐Hispanic Blacks exhibited an overall lower incidence rate of 1.82 (95% CI, 1.76–1.89) over the 2000–2019 period, although the AAPC was not significant at 0.58 (95% CI, −0.27 to 1.53). Hispanics displayed an overall incidence rate of 1.76 (95% CI, 1.70–1.82) from 2000–2019, with an AAPC of −0.02 (95% CI, −0.46 to 0.51) (Table 2).

Non‐Hispanic Whites had the highest incidence rate and the most significant upward trend, whereas non‐Hispanic Blacks had a lower initial rate and a moderate nonsignificant increase in incidence. Hispanics, on the other hand, showed a nonsignificant decreasing trend in vulvar cancer incidence rates over the studied period.

### 3.3 Incidence Trends by Histological Subtypes

The highest age‐adjusted incidence rate of vulvar cancer in the United States over 2000–2019 was for SCC (1.82; 95% CI, 1.80–1.84), followed by other types of vulvar cancer (0.39; 95% CI, 0.38–0.40), BCC (0.15; 95% CI, 0.15–0.16), and adenocarcinoma (0.04; 95% CI, 0.04–0.04) (Table 2). Over 2000–2019, concerning adenocarcinoma, there was a nonsignificant decreasing trend in non‐Hispanic Whites (AAPC: −1.17; 95% CI, −2.96 to 0.63), but it nonsignificantly increased in Hispanics (AAPC: 0.23; 95% CI, −3.81 to 5.38) and had no significant changes in non‐Hispanic Blacks (AAPC: 1.15; 95% CI, −3.23 to 7.03) (Figure S1). There were no significant changes in the age‐adjusted incidence rates of adenocarcinoma by age group (Figure S2).

The age‐adjusted incidence rate of vulvar cancer SCC over 2000–2019 in the United States was highest and lowest among non‐Hispanic Whites and Hispanics with 2.13 (95% CI, 2.11–2.16) and 1.31 (95% CI, 1.26–1.37), respectively (Table 2). Over 2000–2019, the age‐adjusted incidence rate of SCC was relatively increasing at a greater rate in non‐Hispanic Whites (AAPC: 1.66; 95% CI, 1.28–2.12) (Figure S3) and the age‐adjusted incidence rates of SCC significantly increased in overall age groups (Figure S4).

The age‐adjusted incidence rate of vulvar cancer BCC over 2000–2019 in the United States was highest and lowest among non‐Hispanic Whites and non‐Hispanic Blacks with 0.17 (95% CI, 0.16–0.18) and 0.06 (95% CI, 0.05–0.07), respectively (Table 2). Over 2000–2019, there was a nonsignificant decreasing trend among non‐Hispanic Whites (AAPC: −0.54; 95% CI, −1.53 to 0.46) and Hispanics (AAPC: 0.45; 95% CI, −1.87 to 3.36); however, it nonsignificantly increased among non‐Hispanic Blacks (AAPC: 0.56; 95% CI, −4.40 to 6.74) (Figure S5). The age‐adjusted incidence rates of BCC showed no significant changes by age (Figure S6).

The age‐adjusted incidence rate of other types of vulvar cancer over 2000–2019 in the United States was highest and lowest among non‐Hispanic Whites and non‐Hispanic Blacks with 0.44 (95% CI, 0.43–0.46) and 0.22 (95% CI, 0.20–0.25), respectively (Table 2). Over 2000–2019, there were nonsignificant decreasing trends for non‐Hispanic Whites (AAPC: −0.12; 95% CI, −0.66 to 0.74), non‐Hispanic Blacks (AAPC: −1.20; 95% CI, −3.67 to 1.48), and Hispanics (AAPC: −0.16; 95% CI, −1.99 to 2.12) (Figure S7). The age‐adjusted incidence rates of other types of vulvar cancer showed no significant changes by overall age (Figure S8).

### 3.4 Effects of COVID‐19 on Incidence Rates of Vulvar Cancer

There was a significant decrease in the age‐standardized incidence rate of vulvar cancer in all races/ethnicities in all age groups (AAPC: −10.15; 95% CI, −15.35 to −4.94) and those aged 55–69 years (AAPC: −13.51; 95% CI, −22.02 to −4.99) and 70–84 years (AAPC: −11.55; 95% CI, −19.93 to −3.17) from 2019 to November 2020 during the COVID‐19 pandemic (Table 3).

**TABLE 3:** Percent change in age‐standardized incidence rates from 2019 to 2020, by race/ethnicity and age, using the November 2022 data submission.

Age group (year)	Race/ethnicity	2019 ASIR (95% CI)	2020 ASIR (95% CI)	AAPC (95% CI)
00–39	Non‐Hispanic White	0.28 (0.21–0.36)	0.3 (0.23–0.39)	8.59 (−32.19 to 49.36)
	Non‐Hispanic Black	0.45 (0.28–0.67)	0.16 (0.07–0.31)	−65.34 (−93.4 to −37.28)
	Hispanic	0.14 (0.08–0.23)	0.11 (0.05–0.18)	−27.06 (−82.09 to 27.98)
	All races	0.25 (0.2–0.3)	0.2 (0.15–0.24)	−21.39 (−44.64 to 1.87)
40–54	Non‐Hispanic White	2.78 (2.43–3.18)	2.7 (2.34–3.1)	−3.06 (−21.61 to 15.49)
	Non‐Hispanic Black	3.17 (2.42–4.08)	2.45 (1.79–3.27)	−22.64 (−52.4 to 7.12)
	Hispanic	1.14 (0.83–1.54)	1.4 (1.05–1.83)	22.53 (−26.79 to 71.84)
	All races	2.24 (2–2.49)	2.18 (1.95–2.43)	−2.62 (−17.39 to 12.15)
55–69	Non‐Hispanic White	7.35 (6.8–7.94)	6.13 (5.63–6.67)	−16.58 (−26.08 to −7.09)
	Non‐Hispanic Black	5.04 (4.02–6.25)	3.95 (3.06–5.02)	−21.75 (−46.97 to 3.48)
	Hispanic	3.38 (2.67–4.21)	3.65 (2.93–4.5)	8.07 (−24.85 to 40.98)
	All races	5.96 (5.57–6.37)	5.15 (4.79–5.54)	−13.51 (−22.02 to −4.99)
70–84	Non‐Hispanic White	14.72 (13.66–15.83)	12.72 (11.75–13.75)	−13.58 (−22.8 to −4.36)
	Non‐Hispanic Black	4.28 (2.9–6.09)	7.31 (5.46–9.57)	70.73 (−5.37 to 146.82)
	Hispanic	11.18 (9.13–13.56)	9.08 (7.28–11.2)	−18.78 (−41.95 to 4.39)
	All races	12.6 (11.79–13.45)	11.14 (10.39–11.93)	−11.55 (−19.93 to −3.17)
85+	Non‐Hispanic White	19.38 (17.12–21.85)	17.62 (15.46–19.99)	−9.09 (−24.94 to 6.76)
	Non‐Hispanic Black	10.74 (6.26–17.2)	8.01 (4.26–13.69)	−25.47 (−79.29 to 28.35)
	Hispanic	11.63 (7.53–17.17)	19.49 (14.16–26.16)	67.5 (−14.72 to 149.73)
	All races	16.84 (15.05–18.78)	16.25 (14.5–18.16)	−3.51 (−18.53 to 11.52)
All ages	Non‐Hispanic White	3.08 (2.94–3.23)	2.75 (2.61–2.89)	−10.83 (−16.98 to −4.67)
	Non‐Hispanic Black	2.05 (1.78–2.35)	1.78 (1.53–2.06)	−12.99 (−30.42 to 4.44)
	Hispanic	1.78 (1.57–2.01)	1.8 (1.6–2.03)	1.55 (−15.8 to 18.89)
	All races	2.58 (2.47–2.68)	2.32 (2.22–2.41)	−10.15 (−15.35 to −4.94)

Abbreviations: AAPC, average annual percent change; ASIR, age‐standardized incidence rate; CI, confidence interval.

### 3.5 Age Patterns of Vulvar Cancer

**FIGURE 2:**
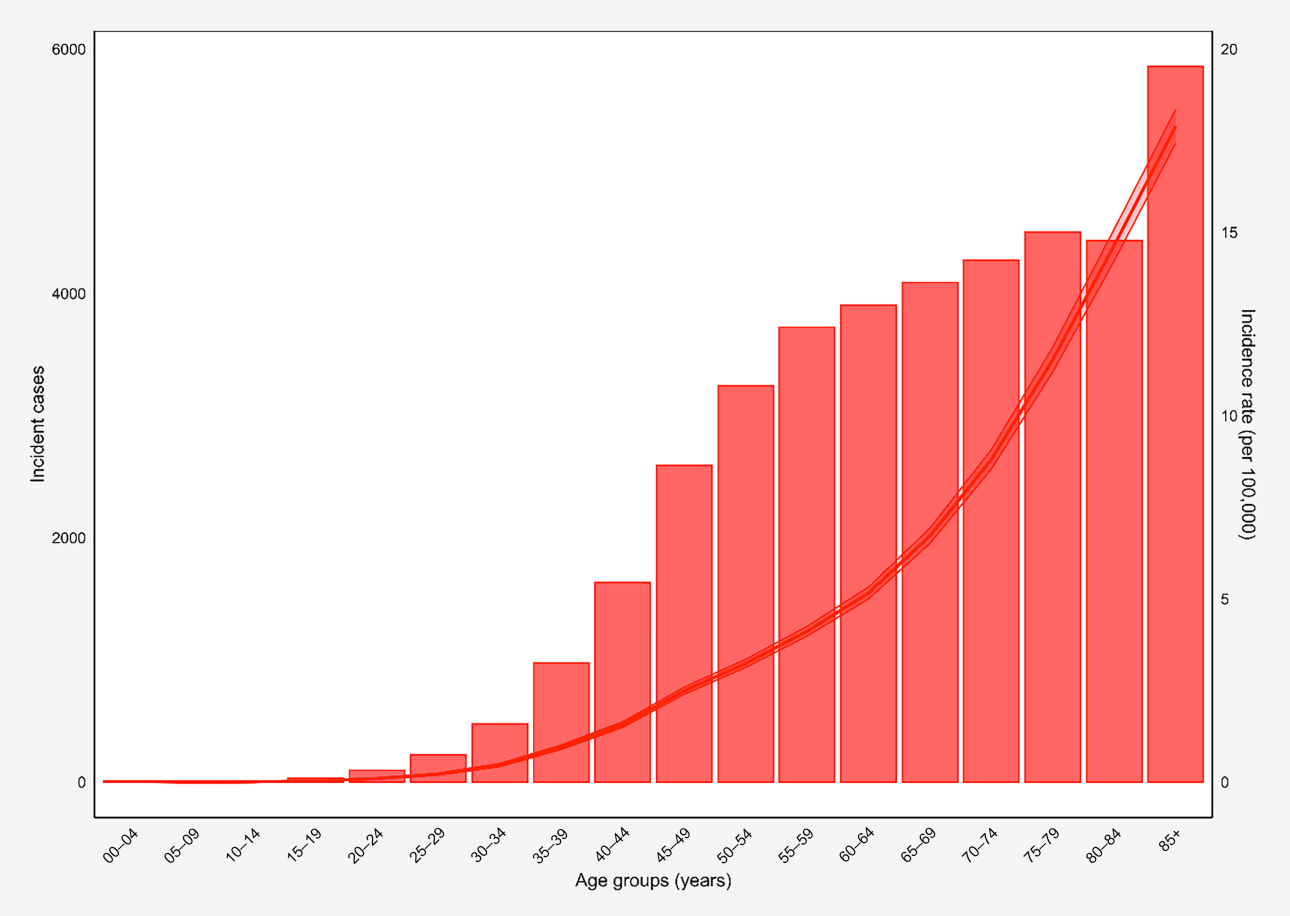
Incident numbers and incidence rate of vulvar cancer in the United States in each age group.

The incidence rates and incident numbers of vulvar cancer increased with aging and peaked in the 85+ age group (Figure 2). Moreover, the incidence rates of adenocarcinoma increased with advancing age. Also, the incident cases of adenocarcinoma peaked in the 60–64 age group (Figure S9). There were also similar trends for incidence rates and incident cases of SCC (Figure S10), BCC (Figure S11), and other types of vulvar cancer (Figure S12).

## 4 Discussion

The primary findings of the current study based on the data from the SEER Program over the years 2000–2019 demonstrated that the incidence rates of vulvar cancer were increasing in the United States. Non‐Hispanic White women had the highest incidence rate for vulvar cancer, with a relatively increasing trend compared to others. Moreover, SCC had the highest incidence rate among vulvar cancer histotypes, with the highest incidence rate and relatively increasing trend in non‐Hispanic Whites compared to other groups.

Our study revealed an increasing incidence rate of vulvar cancer in the United States from 2000 to 2019. In line with our results, a study by Zhou et al. based on the data obtained from the US Cancer Statistics (USCS) database over 2001–2018, indicated an increasing vulvar cancer incidence rate. The same study investigation similarly observed a notable decline in non‐SCC vulvar cancer. However, our study did not identify any statistically significant alterations in histological types apart from SCC. This discrepancy may stem from the differing categorizations. In our study, non‐SCC encompasses adenocarcinoma and BCC, whereas in the other study, non‐SCC includes adenocarcinoma and several distinct histopathologies grouped under “other malignancies” [7]. Furthermore, similar trends regarding overall vulvar cancer incidence rates were discovered in high‐income countries. Another study comprising data from the Saarland Cancer Registry between 1974 and 2013 revealed a significant increase in the vulvar cancer incidence rate in Germany [24]. Likewise, the results of a study retrieved from the United Kingdom Cancer Information Service from 1990–1992 to 2007–2009 showed a similar pattern [25]. According to a previous study in the United States, HPV DNA was detected in almost 69% of patients with vulvar cancer, suggesting a potential link between HPV infection and the incidence of vulvar cancer [26]. However, other global studies normally reported around 30%–40% of vulvar cancers as HPV positive [27]. We theorize that the rising rates of vulvar cancer may be linked to increased HPV exposure, possibly influenced by changes in sexual behavior among women born after 1940, although other factors may also contribute [28, 29]. Moreover, results of a systematic review of 14 studies involving more than 14 000 women showed that vulvar lichen sclerosus elevates the likelihood of vulvar cancer, particularly when combined with differentiated vulvar intraepithelial neoplasia and as age progresses [30]. In this regard, another large‐scale cohort study in the United States highlighted that lichen sclerosis is underrecognition, particularly in younger, fertile women, although obstetrics/gynecologists diagnose and manage about half of these cases [31]. Metabolic factors like diabetes mellitus or high body mass index, as well as immunocompromised states, are also other risk factors for the development of vulvar cancer [32]. The increasing trend of diabetes mellitus globally and in the United States can also explain the increase in incidence rates of vulvar cancer and suggests that maintaining metabolic health can be an option to help prevent vulvar cancer development [33].

HPV vaccination can effectively reduce the incidence of gynecological cancers, particularly cervical and vaginal cancers, with less impact on vulvar cancer due to its lower association with HPV [34]. In this regard, a recently published article by Berenson et al. has found evidence that HPV vaccinations likely contributed to a decrease in the incidences of vulvar carcinoma in situ and invasive vulvar carcinoma among 20‐ to 44‐year‐old women between 2001 and 2018 in the United States [14]. Therefore, some health policy actions like state mandates for HPV vaccination, increasing public awareness about HPV and vulvar cancer using education campaigns, and health insurance support to reduce the costs of HPV vaccination can be carried out in the United States [35].

This congruency of results between our study and those from high‐income countries, such as Germany and the United Kingdom, could also be attributed to diagnostic advances and better access to healthcare. Improved diagnostic techniques and heightened awareness among healthcare providers can lead to earlier and more frequent detection of vulvar cancer cases. This, in turn, can result in an increase in reported incidence rates. In addition, improved access to healthcare services and cancer screening in high‐income countries can result in better case detection and, subsequently, increased incidence rates.

Our study indicated that non‐Hispanic White women had the highest incidence rate of vulvar cancer, with a relatively increasing trend among women of all racial or ethnic groups. Consistent with our results, Zhou et al. reported similar findings [7]. Moreover, Watson et al. conducted a study acquiring data from two federal cancer surveillance programs, representing 92% of the United States population between 1999 and 2004, and declared similar results [36]. Additionally, a study by Saraiya et al. analyzed data representing 83% of the United States population from 1998 to 2003 reported higher incidence rates of vulvar cancer in White women compared to women of other races or ethnicities [10]. The reason for the highest incidence rate of vulvar cancer in non‐Hispanic White women is still unclear. Possible explanations may be attributed to genetic susceptibility in the exposure to some underlying risk factors like HPV positivity, cigarette smoking, first sex before the age of 16 years, and more than one sex partner, which we cannot completely elucidate [37]. Another potential reason could be that vulvar cancer is more common in older women [2], so variations in the age distribution of different racial or ethnic groups could influence the incidence rates. Additionally, genetic determinants could be a possible cause for the higher cancer incidence in White women [38].

Evidence suggests that over 75% of vulvar cancer histotypes comprise SCCs [5, 10]. Our study uncovered that SCC had the highest incidence rate among vulvar cancer histotypes. Notably, the incidence rate of SCC vulvar cancer was highest among non‐Hispanic Whites, with a relatively increasing trend compared to other racial or ethnic groups. In 2018, Van Dyne et al. analyzed data between 1999 and 2015, covering approximately 97.8% of the United States population, and discovered a trend similar to our study among White women [39]. In line with our results, a study by Akhtar‐Danesh et al. using data from the Canadian Cancer Registry dataset between 1992 and 2008 and the American N SEER Program throughout 1973–2010 indicated that the incidence rate of invasive squamous cell vulvar cancer increased in both the United States and Canada [11]. Furthermore, the results from a recent study by Berenson et al., including 88 942 patients with vulvar cancer using the USCS databases from 2001 to 2018, showed that the incidence of vulvar SCC was highest among non‐Hispanic White women, which supports our findings [14]. Prior research has indicated that vulvar SCC risk is linked to anogenital warts, smoking, alcohol use, and socioeconomic factors like marital status and education level [40].

### 4.1 Strengths

The strengths of the current study consist of utilizing a high‐quality, reliable, nationwide, and population‐based database to acquire and analyze data to assess the age‐adjusted incidence rates, APCs, and AAPCs of different vulvar cancer histotypes among diverse racial or ethnic groups in the United States over 20 years from 2000 to 2019. Additionally, widely acknowledged and precise histologic definitions were used to classify various histologic types of vulvar cancer.

### 4.2 Limitations

There were some limitations to the present study. First, we did not estimate the incidence rates, APCs, and AAPCs of vulvar cancer concerning geographical regions. Furthermore, the SEER databases, do not provide the necessary information to report other indicators such as disability‐adjusted life years, years of life lost and years lived with disability. So, we could not report those indicators and this can be considered in the next iteration of the SEER database. Second, we could not evaluate the correlation between vulvar cancer incidence and the relevant risk factors, including HPV status or smoking, since this information is unavailable in the database. Third, there might be minor misclassification regarding patients' race or ethnicity because practitioners conducted the preliminary data collection. Fourth, adenocarcinoma only made up a few cases and rates of the total values.

## 5 Conclusions

The incidence rates of vulvar cancer increased among women from all racial or ethnic groups, with a significantly increased trend in non‐Hispanic Whites from 2000 to 2019. The findings may enable clinicians and healthcare officials to focus more on people from a particular race or ethnicity with a higher incidence rate of vulvar cancer to deliver more optimized and target‐oriented care. We recommend that researchers report the epidemiology of vulvar cancer in the United States by relevant risk factors and update them regularly.

## Author Contributions

**Seyed Ehsan Mousavi:** conceptualization (equal); formal analysis (equal); methodology (equal); resources (equal); software (equal); visualization (equal); writing – original draft (equal); writing – review and editing (equal). **Hoomaan Ghasemi:** writing – original draft (equal); writing – review and editing (equal). **Morvarid Najafi:** writing – original draft (equal); writing – review and editing (equal). **Zahra Yekta:** writing – original draft (equal); writing – review and editing (equal). **Seyed Aria Nejadghaderi:** conceptualization (equal); project administration (equal); resources (equal); supervision (equal); validation (equal); writing – original draft (equal); writing – review and editing (equal).

## Conflicts of Interest

The authors declare no conflicts of interest.

## Disclosure

This study is based on the available data and solely reflects the opinions of its authors and not that of the National Cancer Institute.

## Supporting information


Data S1.

Data S2.

## Data Availability

The data presented in this study are available at https://seer.cancer.gov/data‐software/.

## References

[1] F. B. Stehman and K. Y. Look , “Carcinoma of the Vulva,” Obstetrics and Gynecology 107, no. 3 (2006): 719–733. 16507947 10.1097/01.AOG.0000202404.55215.72

[2] J. M. Mix , S. V. Gopalani , S. Simko , and M. Saraiya , “Trends in HPV‐ and Non‐HPV‐Associated Vulvar Cancer Incidence, United States, 2001–2017,” Preventive Medicine 164 (2022): 107302. 36240909 10.1016/j.ypmed.2022.107302 PMC10999169

[3] R. L. Siegel , K. D. Miller , N. S. Wagle , and A. Jemal , “Cancer Statistics, 2023,” Cancer Journal for Clinicians 73, no. 1 (2023): 17–48. 10.3322/caac.21763 36633525

[4] American Cancer Society , “Cancer Types,” 2023, https://www.cancer.org/cancer/types/vulvar‐cancer/detection‐diagnosis‐staging/survival‐rates.html.

[5] M. S. Schuurman , L. C. van den Einden , L. F. Massuger , L. A. Kiemeney , M. A. van der Aa , and J. A. de Hullu , “Trends in Incidence and Survival of Dutch Women With Vulvar Squamous Cell Carcinoma,” European Journal of Cancer 49, no. 18 (2013): 3872–3880. 24011936 10.1016/j.ejca.2013.08.003

[6] A. Capria , N. Tahir , and M. Fatehi , “Vulva Cancer,” in StatPearls [Internet] (Treasure Island, FL: StatPearls Publishing, 2024), https://www.ncbi.nlm.nih.gov/books/NBK567798/. 33620867

[7] W.‐L. Zhou and Y.‐Y. Yue , “Trends in the Incidence of Vulvar and Vaginal Cancers With Different Histology by Race, Age, and Region in the United States (2001–2018),” International Journal of Public Health 67 (2022). 10.3389/ijph.2022.1605021 PMC9464823 36105176

[8] J. A. Rauh‐Hain , J. Clemmer , R. M. Clark , et al., “Racial Disparities and Changes in Clinical Characteristics and Survival for Vulvar Cancer Over Time,” American Journal of Obstetrics and Gynecology 209, no. 5 (2013): 468.e1‐e10. 10.1016/j.ajog.2013.07.021 23891626

[9] C.‐I. Liao , A. Moon , K. Lin , et al., “Racial Disparities in the Incidence of Vulvar Cancer in the United States: A Study of 75,767 Over 16 Years,” Gynecologic Oncology 162 (2021): S251.

[10] M. Saraiya , M. Watson , X. Wu , et al., “Incidence of In Situ and Invasive Vulvar Cancer in the US, 1998–2003,” Cancer 113, no. S10 (2008): 2865–2872. 18980209 10.1002/cncr.23759

[11] N. Akhtar‐Danesh , L. Elit , and A. Lytwyn , “Trends in Incidence and Survival of Women With Invasive Vulvar Cancer in the United States and Canada: A Population‐Based Study,” Gynecologic Oncology 134, no. 2 (2014): 314–318. 24875124 10.1016/j.ygyno.2014.05.014

[12] X. He , Q. Dong , C. Weng , J. Gu , Q. Yang , and G. Yang , “Trends in Incidence, Survival and Initial Treatments of Gynecological Sarcoma: A Retrospective Analysis of the United States Subpopulation,” BioMed Central Womens Health 23, no. 1 (2023): 10. 10.1186/s12905-023-02161-1 PMC9830743 36624439

[13] J. W. Lee , Y. T. Ouh , H. K. Chang , et al., “Trends in Gynecologic Carcinosarcoma Based on Analysis of the Surveillance Epidemiology End Result (SEER) Database,” Journal of Clinical Medicine 12, no. 3 (2023). 10.3390/jcm12031188 PMC9917500 36769835

[14] A. B. Berenson , M. Chang , E. T. Hawk , L. M. Ramondetta , and T. Hoang , “Vulvar Cancer Incidence in the United States and Its Relationship to Human Papillomavirus Vaccinations, 2001–2018,” Cancer Prevention Research 15, no. 11 (2022): 777–784. 35969832 10.1158/1940-6207.CAPR-22-0086

[15] SEER , “About the SEER Program—SEER,” 2023, https://seer.cancer.gov/about/overview.html.

[16] The National Cancer Institute's SEER 22 Databse , “Surveillance, Epidemiology, and End Results (SEER) Program, SEER*Stat Database: Incidence—SEER Research Limited‐Field Data, 22 Registries, November 2021 Sub (2000–2019)—Linked to County Attributes—Time Dependent (1990–2019) Income/Rurality, 1969–2020 Counties, National Cancer Institute, DCCPS, Surveillance Research Program, Released April 2022, Based on the November 2021 Submission,”, https://www.seer.cancer.gov.

[17] SEER , “SEER Research Data Agreement,” accessed April 11, 2023, https://seer.cancer.gov/data‐software/documentation/seerstat/nov2021/seer‐dua‐nov2021.html.

[18] SEER , “Surveillance Research Program, National Cancer Institute SEER*Stat Software,” Version 4.8.1, https://www.seer.cancer.gov/seerstat.

[19] R. C. Tiwari , L. X. Clegg , and Z. Zou , “Efficient Interval Estimation for Age‐Adjusted Cancer Rates,” Statistical Methods in Medical Research 15, no. 6 (2006): 547–569. 17260923 10.1177/0962280206070621

[20] National Cancer Institute , Joinpoint Regression Program, Version 4.9.1.0 (Statistical Methodology and Applications Branch, Surveillance Research Program, National Cancer Institute, United States, April 2022).

[21] H. J. Kim , M. P. Fay , E. J. Feuer , and D. N. Midthune , “Permutation Tests for Joinpoint Regression With Applications to Cancer Rates,” Statistics in Medicine 19, no. 3 (2000): 335–351. 10649300 10.1002/(sici)1097-0258(20000215)19:3<335::aid-sim336>3.0.co;2-z

[22] A. B. Mariotto , E. J. Feuer , N. Howlader , H.‐S. Chen , S. Negoita , and K. A. Cronin , “Interpreting Cancer Incidence Trends: Challenges Due to the COVID‐19 Pandemic,” Journal of the National Cancer Institute 115, no. 9 (2023): 1109–1111. 37220901 10.1093/jnci/djad086 PMC10483261

[23] H. J. Kim , M. P. Fay , B. Yu , M. J. Barrett , and E. J. Feuer , “Comparability of Segmented Line Regression Models,” Biometrics 60, no. 4 (2004): 1005–1014. 15606421 10.1111/j.0006-341X.2004.00256.x

[24] B. Holleczek , J. Sehouli , and J. Barinoff , “Vulvar Cancer in Germany: Increase in Incidence and Change in Tumour Biological Characteristics From 1974 to 2013,” Acta Oncologica 57, no. 3 (2018): 324–330. 28799431 10.1080/0284186X.2017.1360513

[25] J. Lai , R. Elleray , A. Nordin , et al., “Vulval Cancer Incidence, Mortality and Survival in England: Age‐Related Trends,” BJOG: An International Journal of Obstetrics & Gynaecology 121, no. 6 (2014): 728–738. 24148762 10.1111/1471-0528.12459

[26] M. Saraiya , E. R. Unger , T. D. Thompson , et al., “US Assessment of HPV Types in Cancers: Implications for Current and 9‐Valent HPV Vaccines,” Journal of the National Cancer Institute 107, no. 6 (2015): djv086. 25925419 10.1093/jnci/djv086 PMC4838063

[27] S. de Sanjosé , L. Alemany , J. Ordi , et al., “Worldwide Human Papillomavirus Genotype Attribution in Over 2000 Cases of Intraepithelial and Invasive Lesions of the Vulva,” European Journal of Cancer 49, no. 16 (2013): 3450–3461. 23886586 10.1016/j.ejca.2013.06.033

[28] G. Liu , S. Hariri , H. Bradley , S. L. Gottlieb , J. S. Leichliter , and L. E. Markowitz , “Trends and Patterns of Sexual Behaviors Among Adolescents and Adults Aged 14 to 59 Years, United States,” Sexually Transmitted Diseases 42, no. 1 (2015): 20–26. 25504296 10.1097/OLQ.0000000000000231 PMC6785975

[29] V. Sigusch , “On Cultural Transformations of Sexuality and Gender in Recent Decades,” German Medical Science 2 (2004): Doc07. 19675690 PMC2703209

[30] P. Vieira‐Baptista , F. R. Pérez‐López , M. T. López‐Baena , C. K. Stockdale , M. Preti , and J. Bornstein , “Risk of Development of Vulvar Cancer in Women With Lichen Sclerosus or Lichen Planus: A Systematic Review,” Journal of Lower Genital Tract Disease 26, no. 3 (2022): 250–257. 35285455 10.1097/LGT.0000000000000673

[31] L. E. Melnick , A. B. Steuer , A. K. Bieber , P. W. Wong , and M. K. Pomeranz , “Lichen Sclerosus Among Women in the United States,” International Journal of Women's Dermatology 6, no. 4 (2020): 260–262. 10.1016/j.ijwd.2020.05.001 PMC7522895 33015282

[32] L. Bucchi , M. Pizzato , S. Rosso , and S. Ferretti , “New Insights Into the Epidemiology of Vulvar Cancer: Systematic Literature Review for an Update of Incidence and Risk Factors,” Cancers 14, no. 2 (2022). 10.3390/cancers14020389 PMC8773873 35053552

[33] S. Safiri , N. Karamzad , J. S. Kaufman , et al., “Prevalence, Deaths and Disability‐Adjusted‐Life‐Years (DALYs) due to Type 2 Diabetes and Its Attributable Risk Factors in 204 Countries and Territories, 1990–2019: Results From the Global Burden of Disease Study 2019,” Frontiers in Endocrinology 13 (2022). 10.3389/fendo.2022.838027 PMC8915203 35282442

[34] K. S. Kechagias , I. Kalliala , S. J. Bowden , et al., “Role of Human Papillomavirus (HPV) Vaccination on HPV Infection and Recurrence of HPV Related Disease After Local Surgical Treatment: Systematic Review and Meta‐Analysis,” British Medical Journal 378 (2022): e070135. 35922074 10.1136/bmj-2022-070135 PMC9347010

[35] J. Hirth , “Disparities in HPV Vaccination Rates and HPV Prevalence in the United States: A Review of the Literature,” Human Vaccines & Immunotherapeutics 15, no. 1 (2019): 146–155. 30148974 10.1080/21645515.2018.1512453 PMC6363146

[36] M. Watson , M. Saraiya , and X. Wu , “Update of HPV‐Associated Female Genital Cancers in the United States, 1999–2004,” Journal of Women's Health 18, no. 11 (2009): 1731–1738. 10.1089/jwh.2009.1570 19951205

[37] L. Lin , V. B. Benard , A. Greek , N. A. Hawkins , K. B. Roland , and M. Saraiya , “Racial and Ethnic Differences in Human Papillomavirus Positivity and Risk Factors Among Low‐Income Women in Federally Qualified Health Centers in the United States,” Preventive Medicine 81 (2015): 258–261. 26361751 10.1016/j.ypmed.2015.08.027 PMC4751981

[38] B. C. Özdemir and G. P. Dotto , “Racial Differences in Cancer Susceptibility and Survival: More Than the Color of the Skin?” Trends in Cancer 3, no. 3 (2017): 181–197. 28718431 10.1016/j.trecan.2017.02.002 PMC5518637

[39] E. A. Van Dyne , S. J. Henley , M. Saraiya , C. C. Thomas , L. E. Markowitz , and V. B. Benard , “Trends in Human Papillomavirus‐Associated Cancers—United States, 1999–2015,” MMWR. Morbidity and Mortality Weekly Report 67, no. 33 (2018): 918–924. 30138307 10.15585/mmwr.mm6733a2 PMC6107321

[40] B. S. Madsen , H. L. Jensen , A. J. van den Brule , J. Wohlfahrt , and M. Frisch , “Risk Factors for Invasive Squamous Cell Carcinoma of the Vulva and Vagina—Population‐Based Case‐Control Study in Denmark,” International Journal of Cancer 122, no. 12 (2008): 2827–2834. 18348142 10.1002/ijc.23446

