# Supplementary material for: Incidence Trends of Vulvar Cancer in the United States: A 20‐Year Population‐Based Study
Source: Cancer Rep (Hoboken). 2024 Jun 21;7(6):e2120. doi: 10.1002/cnr2.2120 (PMC11190582; doi:10.1002/cnr2.2120)
Supplement: Supplementary file 2 — Data S2. [file CNR2-7-e2120-s001.docx]

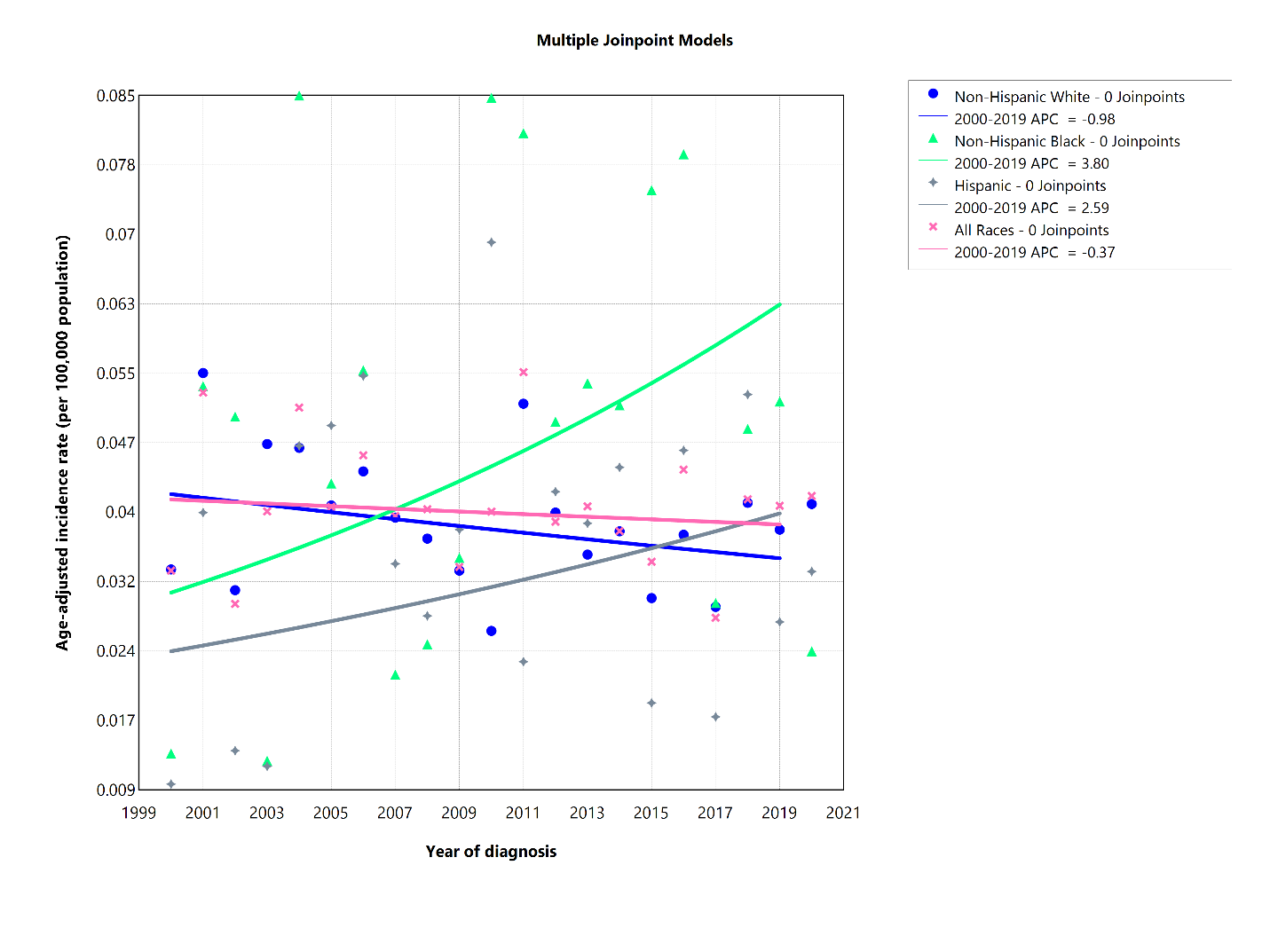


**Figure S1.** Age-adjusted incidence rate of adenocarcinoma per 100,000 people over 2000-2019 and in 2020 in the United States, by race/ethnicity. APC: annual percent change.


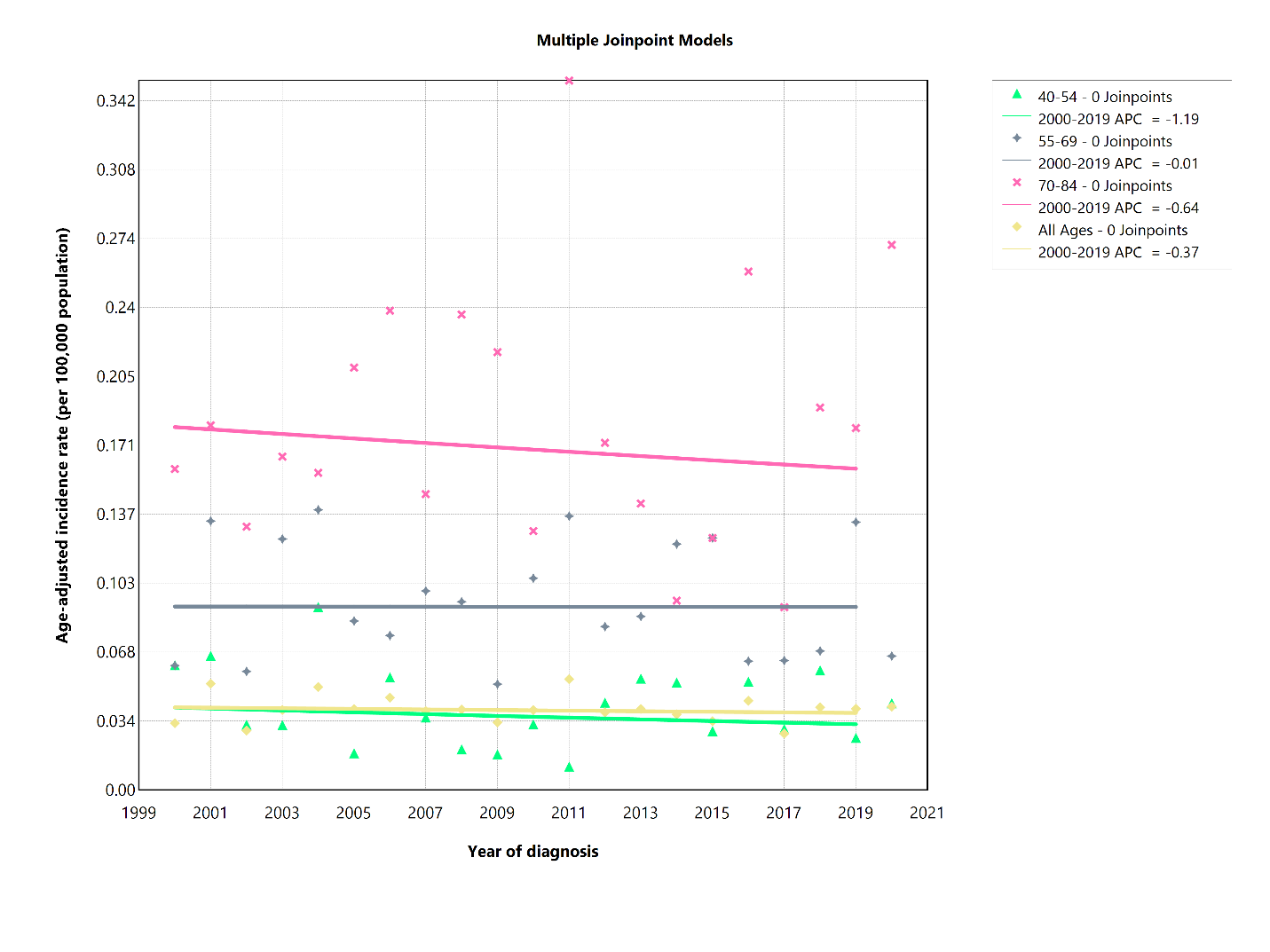


**Figure S2.** Age-adjusted incidence rate of adenocarcinoma over 2000-2019 and in 2020 in the United States, by age. APC: annual percent change.


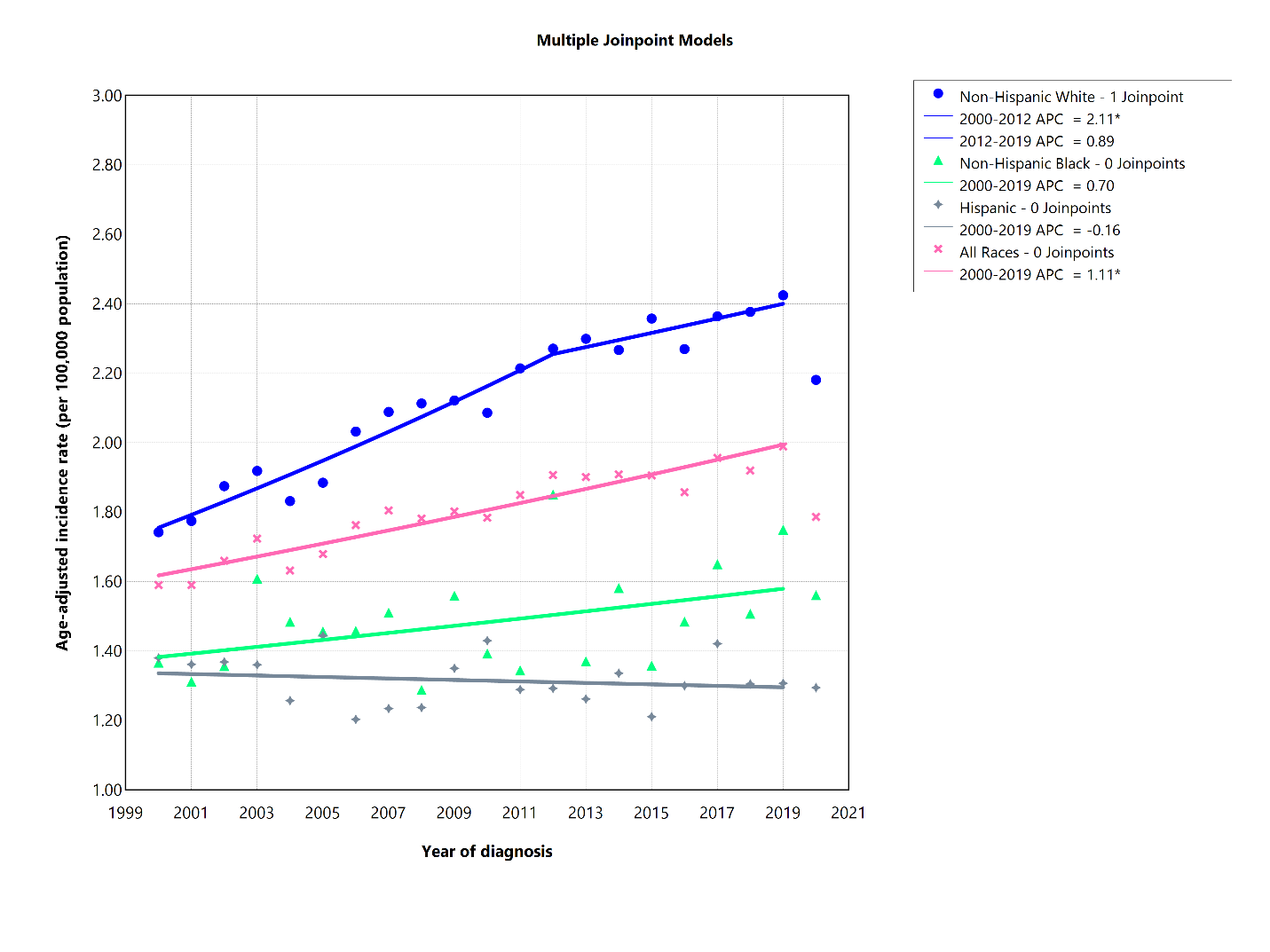


**Figure S3.** Age-adjusted incidence rate of squamous cell carcinoma per 100,000 people over 2000-2019 and in 2020 in the United States, by race/ethnicity. APC: annual percent change. * Represent p-value less than 0.05.


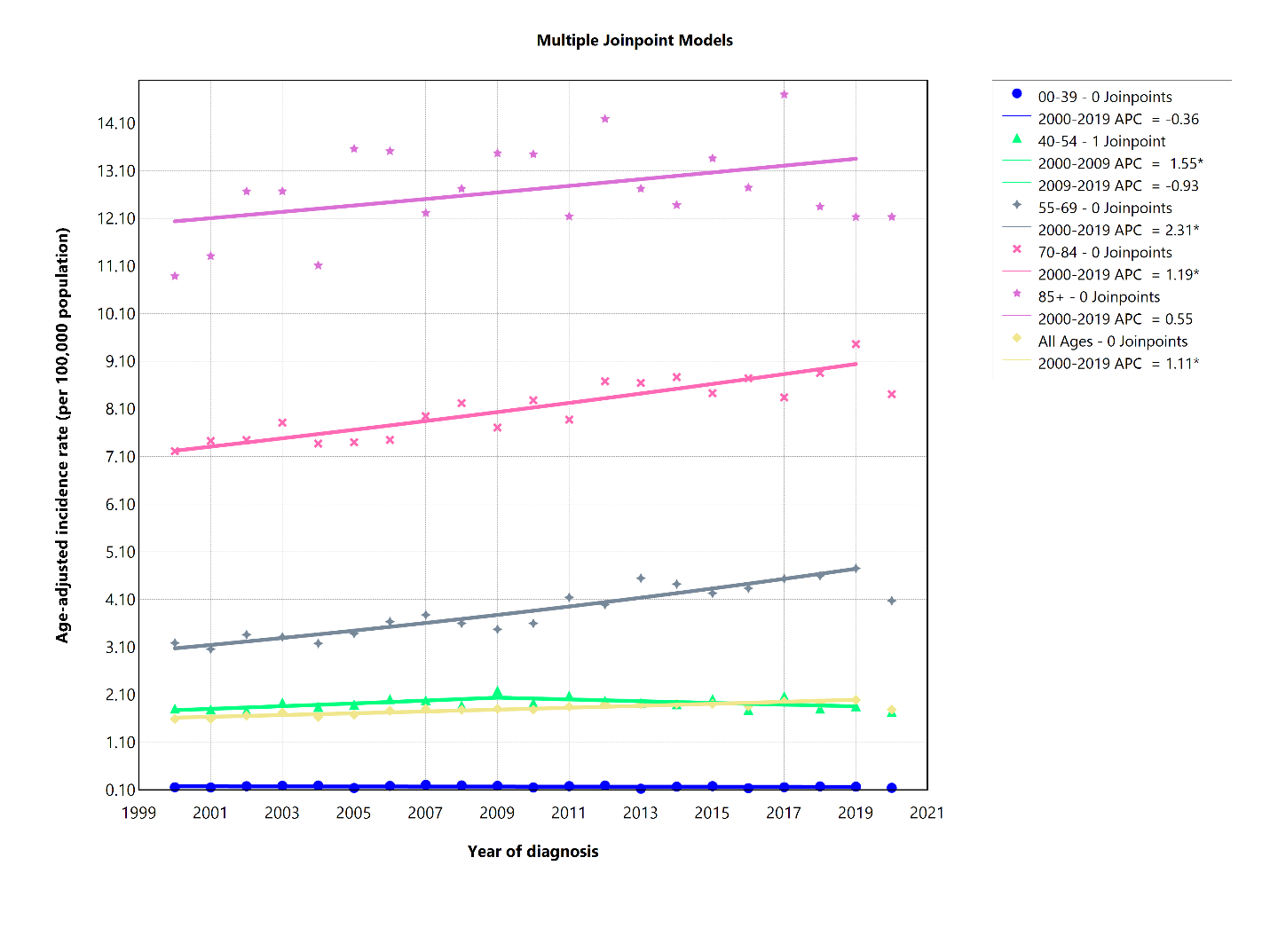


**Figure S4.** Age-adjusted incidence rate of squamous cell carcinoma per 100,000 people over 2000-2019 and in 2020 in the United States, by age. APC: annual percent change. * Represent p-value less than 0.05.


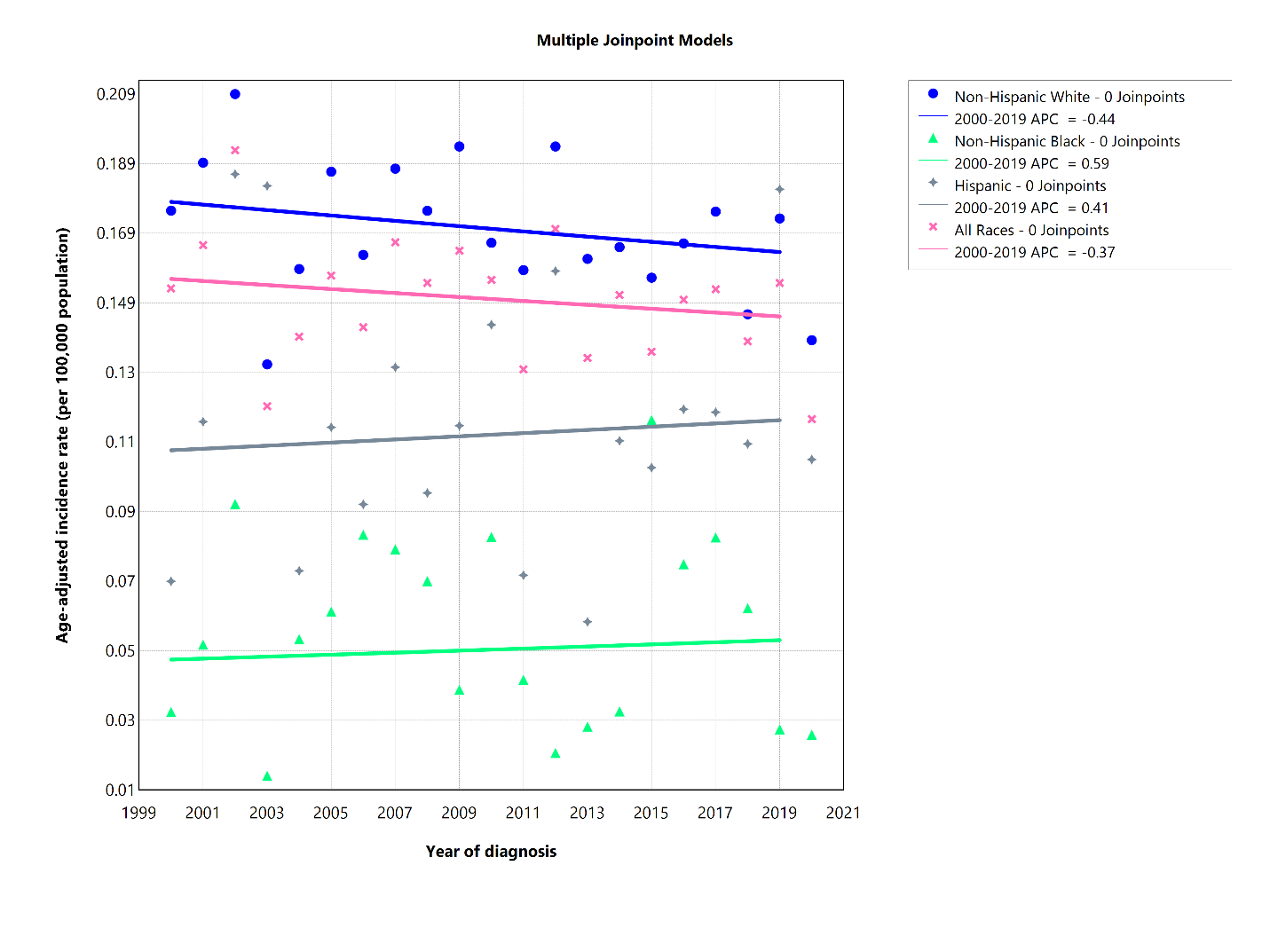


**Figure S5.** Age-adjusted incidence rate of basal cell carcinoma per 100,000 people over 2000-2019 and in 2020 in the United States, by race/ethnicity. APC: annual percent change.


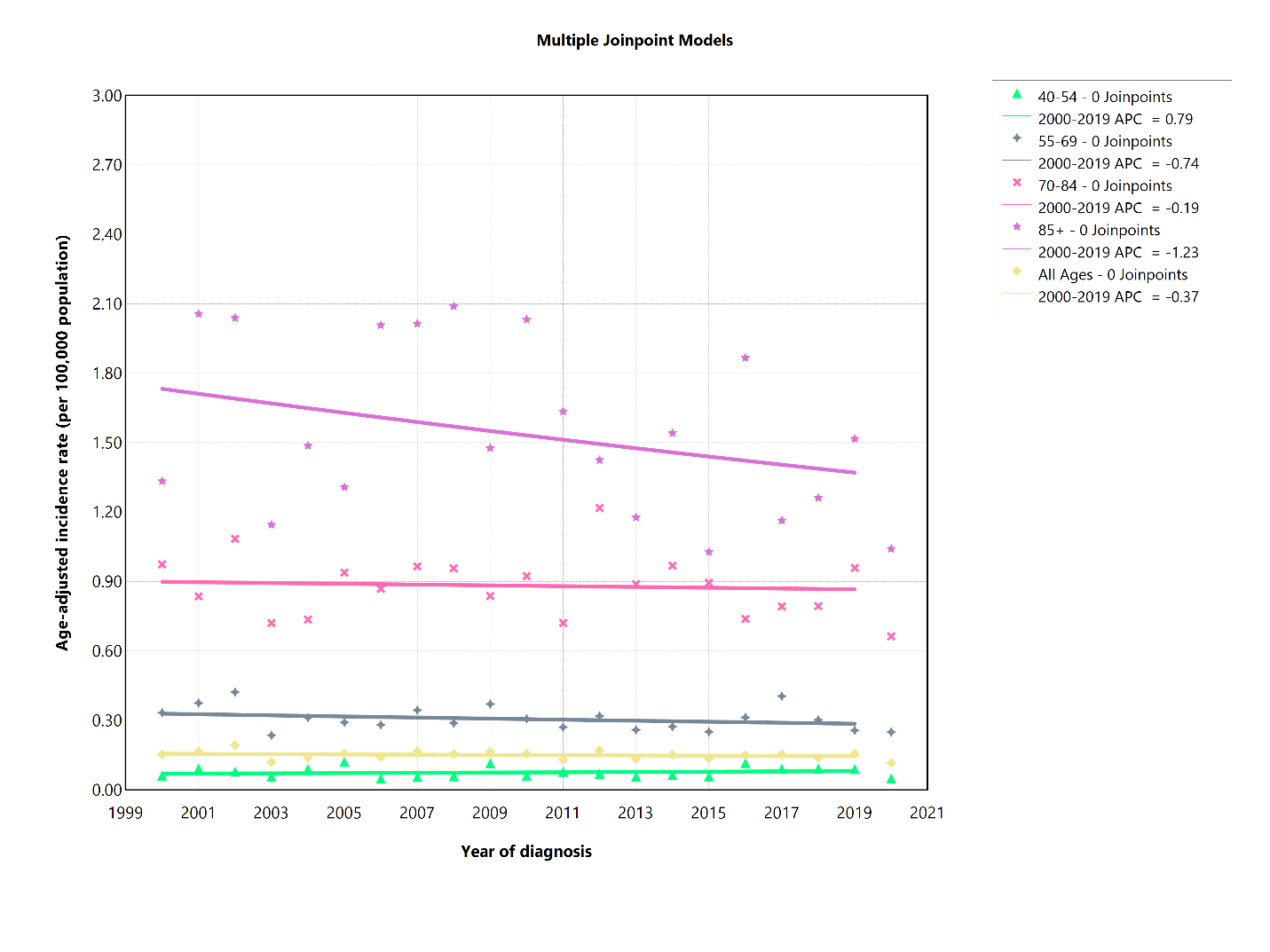


**Figure S6.** Age-adjusted incidence rate of basal cell carcinoma per 100,000 people over 2000-2019 and in 2020 in the United States, by age. APC: annual percent change.


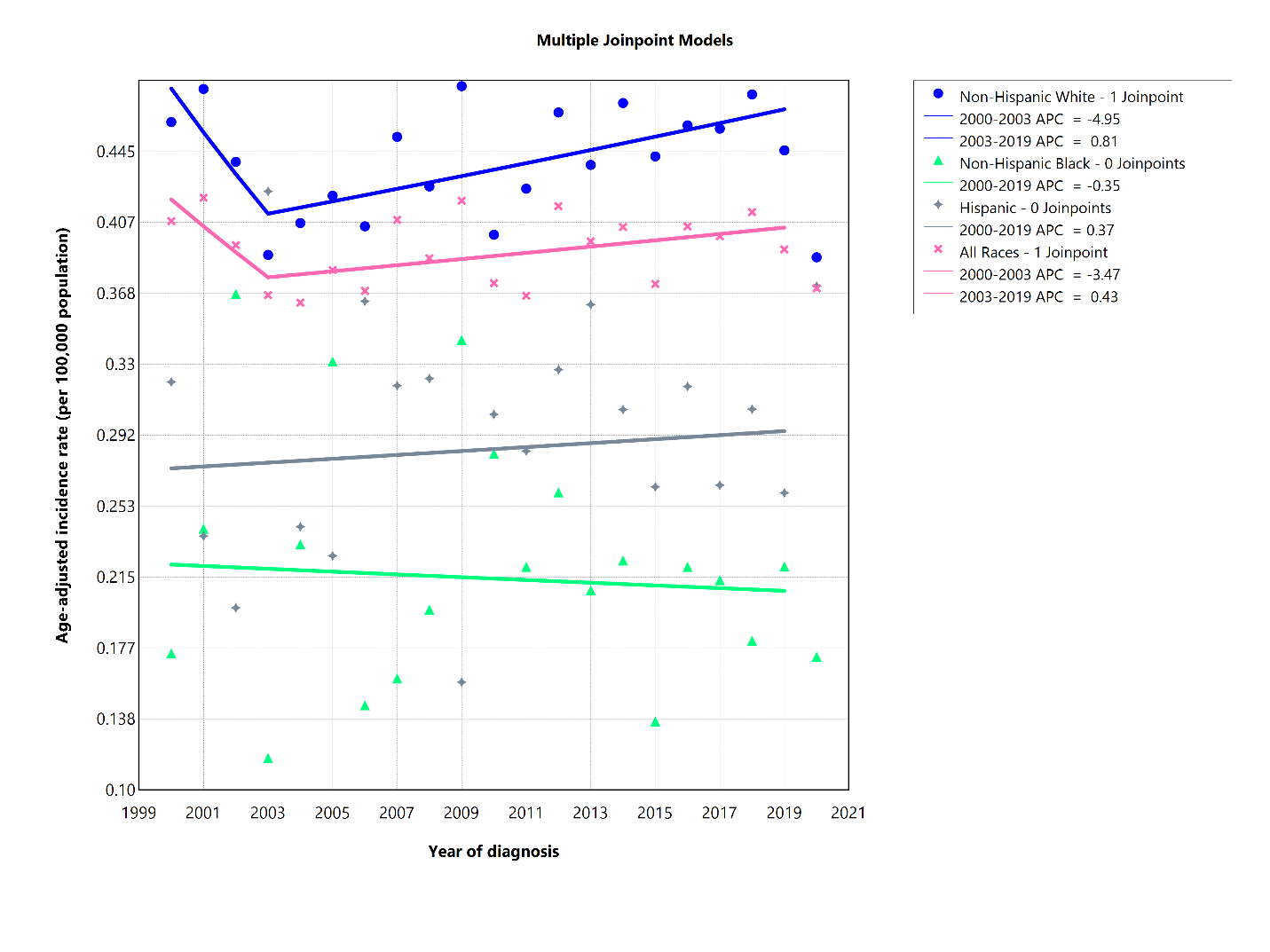


**Figure S7.** Age-adjusted incidence rate of other cancers per 100,000 people over 2000-2019 and in 2020 in the United States, by race/ethnicity. APC: annual percent change.

**
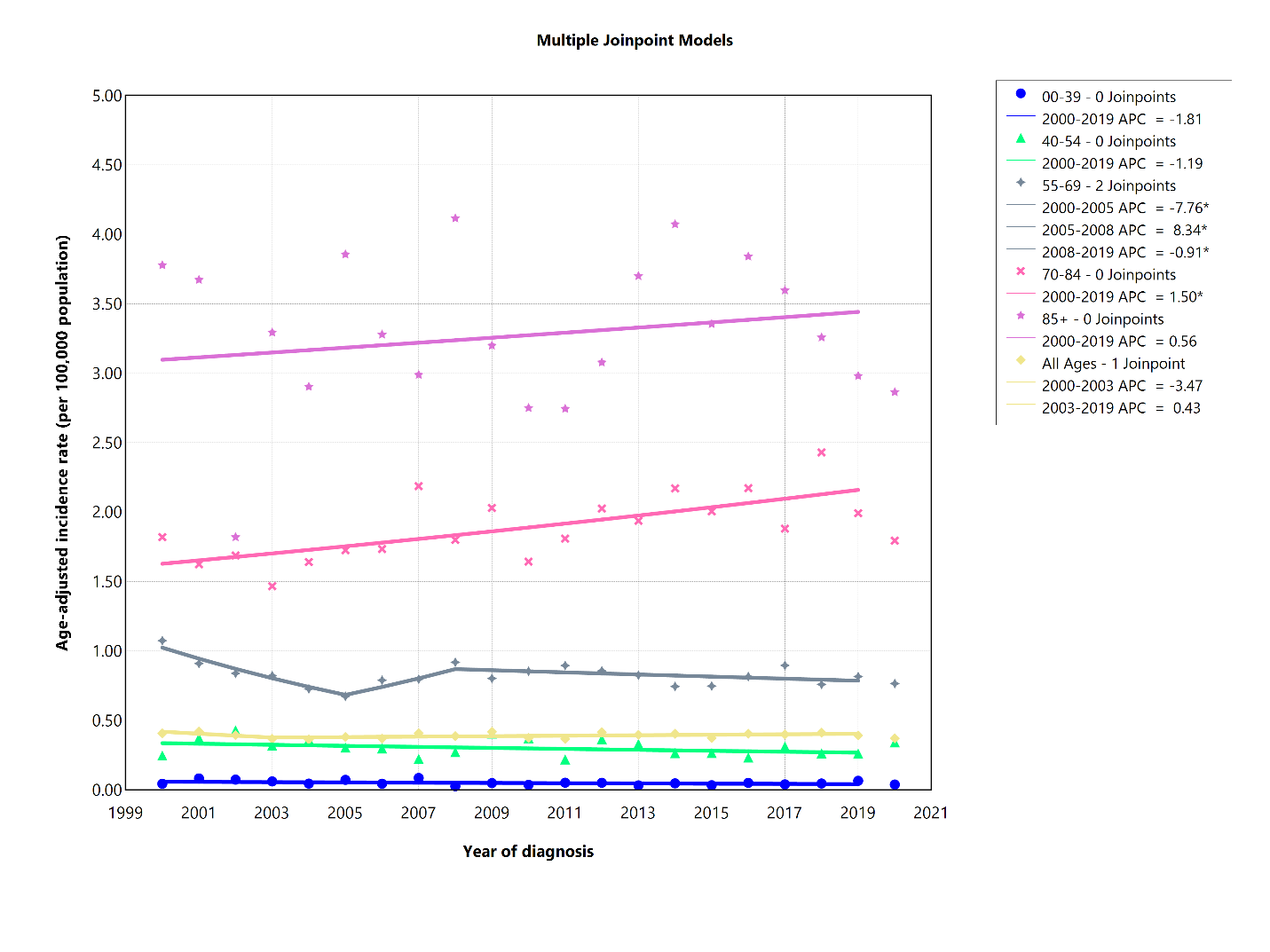
**

**Figure S8.** Age-adjusted incidence rate of other cancers per 100,000 people over 2000-2019 and in 2020 in the United States, by age. APC: annual percent change. * Represent p-value less than 0.05.


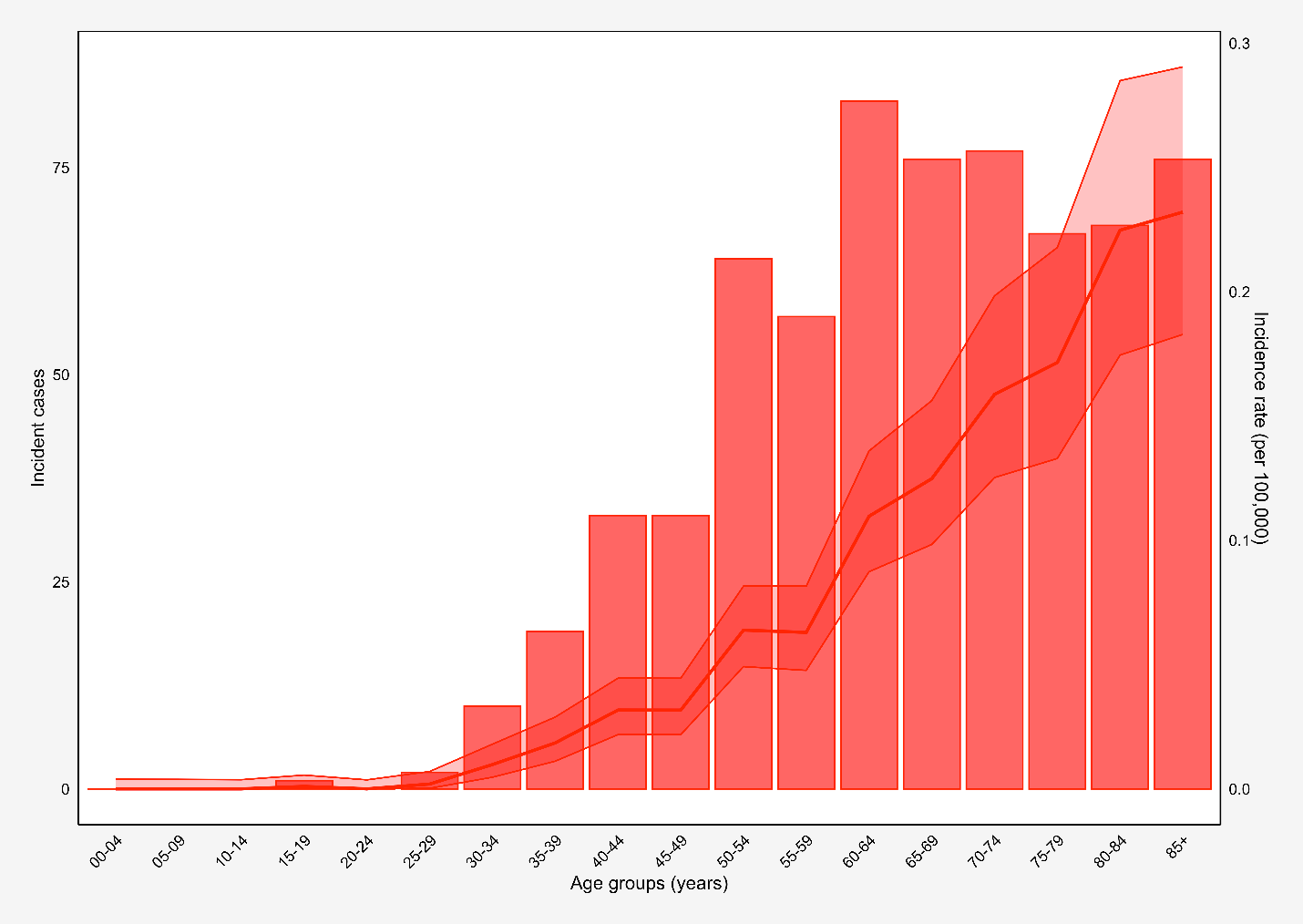


**Figure S9.** Incident numbers and age-adjusted incidence rate of adenocarcinoma in the United States in each age group.

**
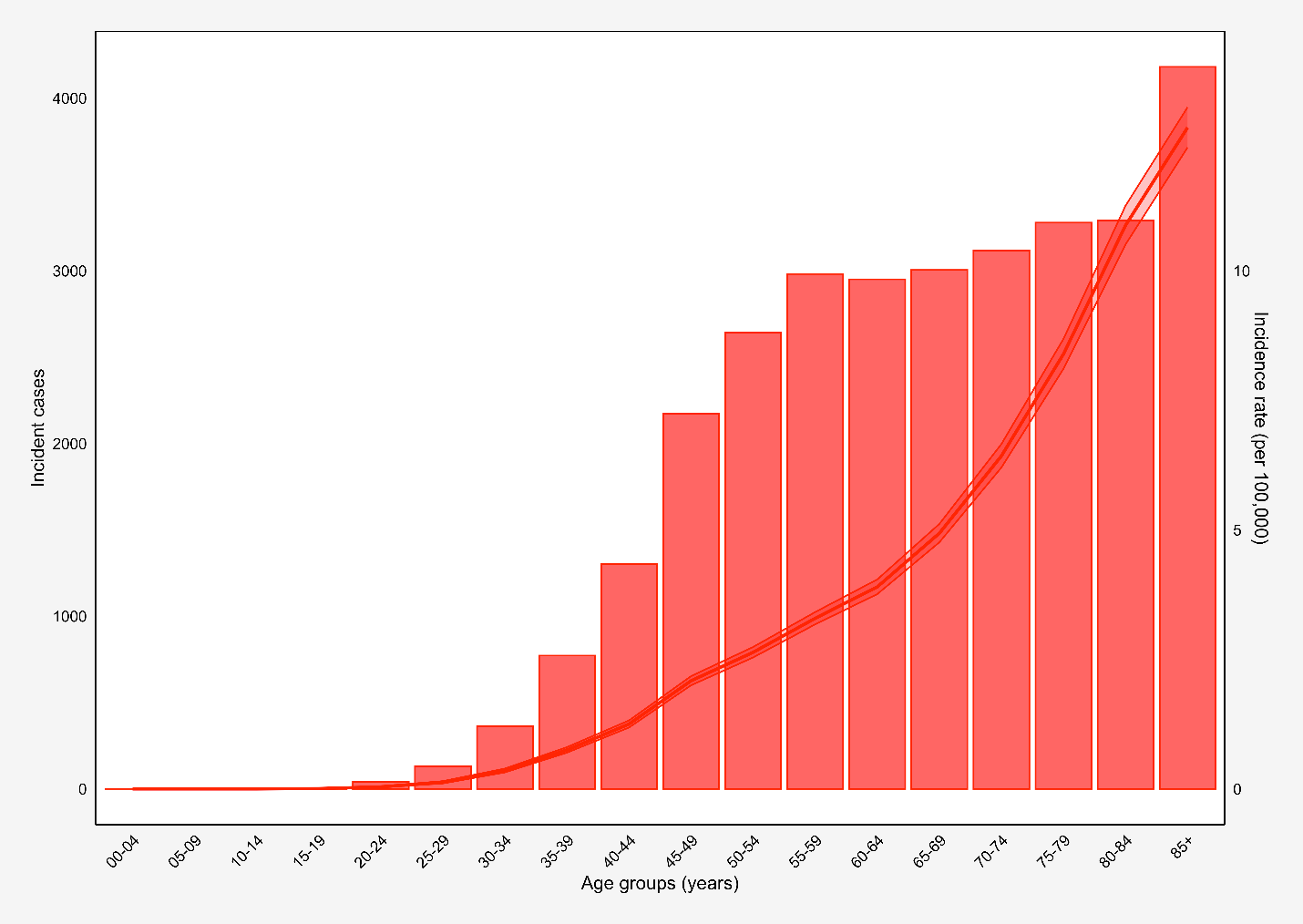
**

**Figure S10.** Incident numbers and age-adjusted incidence rate of squamous cell carcinoma in the United States in each age group.

**
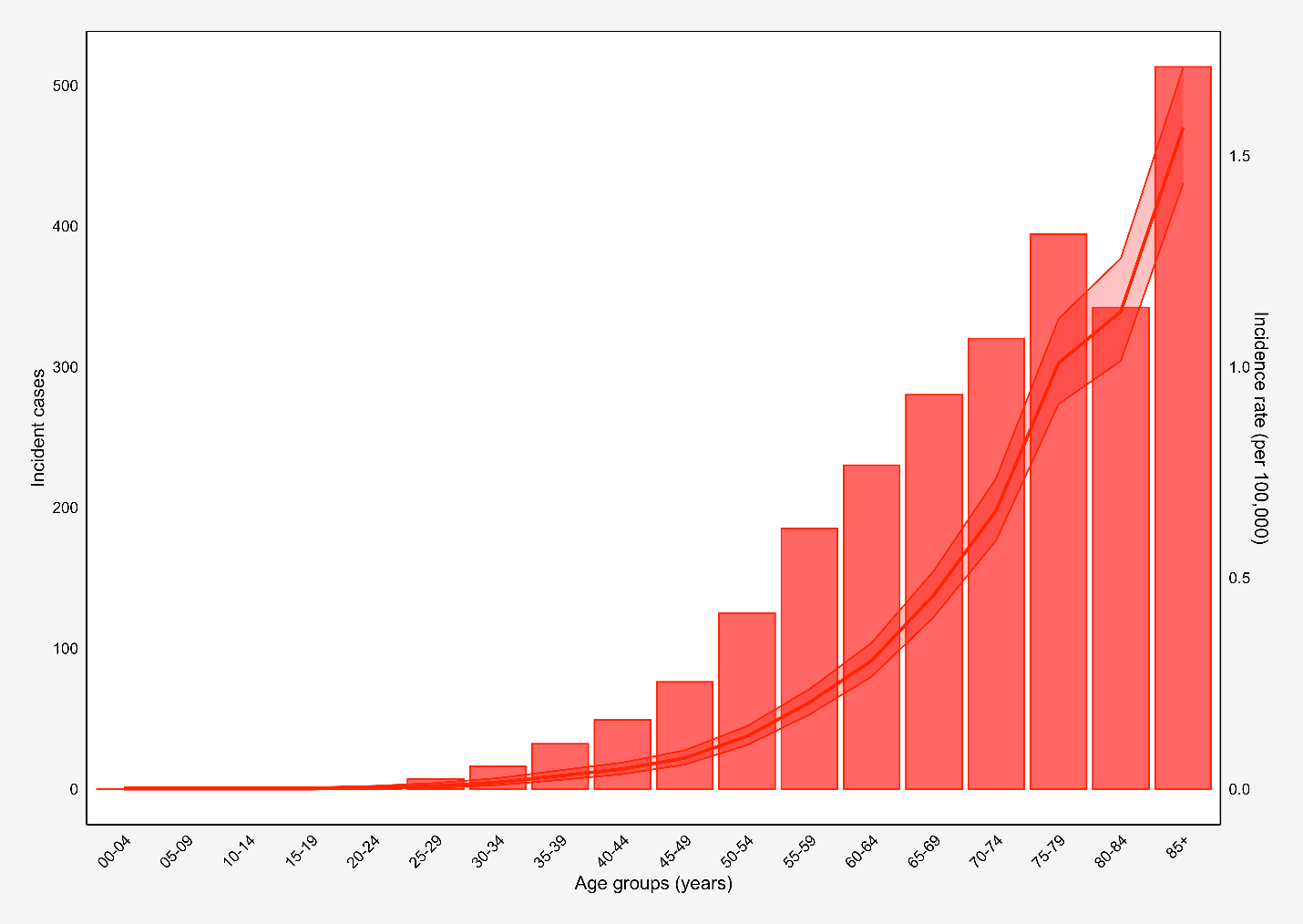
**

**Figure S11.** Incident numbers and age-adjusted incidence rate of basal cell carcinoma in the United States in each age group.


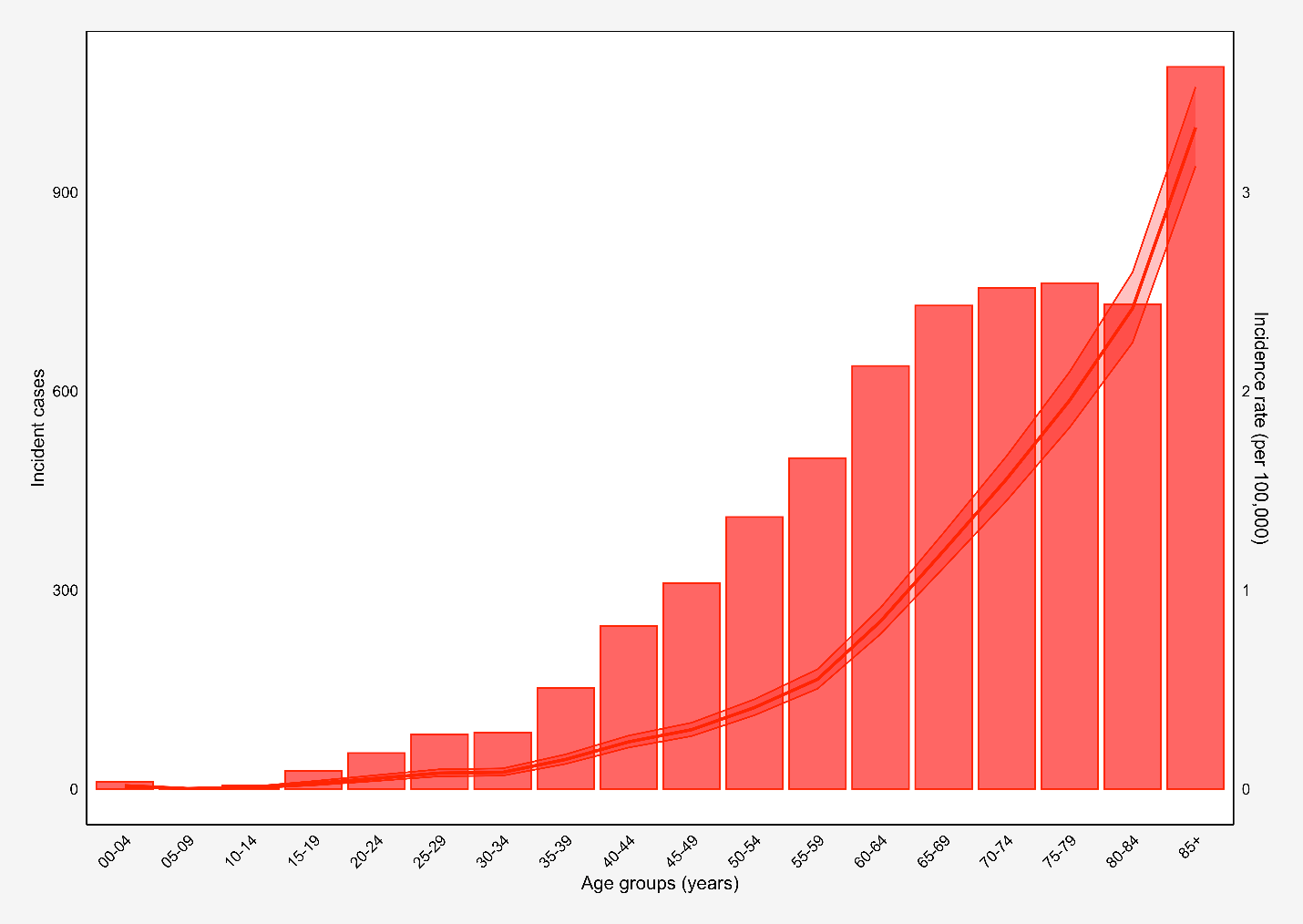


**Figure S12.** Incident numbers and age-adjusted incidence rate of other cancers in the United States in each age group.
